# Targeted Physical Therapy Combined with Spasticity Management Changes Motor Development Trajectory for a 2-Year-Old with Cerebral Palsy

**DOI:** 10.3390/jpm11030163

**Published:** 2021-02-27

**Authors:** Corri L. Stuyvenberg, Shaaron E. Brown, Ketaki Inamdar, Megan Evans, Lin-ya Hsu, Olivier Rolin, Regina T. Harbourne, Sarah Westcott McCoy, Michele A. Lobo, Natalie A. Koziol, Stacey C. Dusing

**Affiliations:** 1 Rehabilitation Science Graduate Program, University of Minnesota Medical School, MMC 388, 420 Delaware St. SE, Minneapolis, MN 55455, USA; stuyv006@umn.edu; 2 Department of Physical Therapy, Virginia Commonwealth University Health System, 1300 East Marshall Street, P.O. Box 980419, Richmond, VA 23298, USA; shaaron.brown@vcuhealth.org; 3 Rehabilitation and Movement Science Program, College of Health Professions, Virginia Commonwealth University, 900 E. Leigh Street, Richmond, VA 23298, USA; inamdark@mymail.vcu.edu; 4 College of Health Professions, Virginia Commonwealth University, 900 E. Leigh Street, Richmond, VA 23298, USA; evansm7@vcu.edu; 5 Department of Rehabilitation Medicine, Division of Physical Therapy, University of Washington, 1959 NE Pacific Street, P.O. Box 356490, Seattle, WA 98195, USA; linyahsu@uw.edu (L.-y.H.); westcs@uw.edu (S.W.M.); 6 Department of Physical Medicine and Rehabilitation, Virginia Commonwealth University Health Sciences, 1223 E. Marshall Street, P.O. Box 980677, Richmond, VA 23298, USA; olivier.rolin@vcuhealth.org; 7 Rangos School of Health Sciences, Physical Therapy, Duquesne University, 600 Forbes Ave., Pittsburgh, PA 15282, USA; harbourner@duq.edu; 8 Department of Physical Therapy and Biomechanics & Movement Science Program, University of Delaware, 540 S. College Ave., Newark, DE 19711, USA; malobo@udel.edu; 9 Nebraska Center for Research on Children, Youth, Families & Schools, University of Nebraska Lincoln, 160 Prem S. Paul Research Center at Whittier School, Lincoln, NE 68583, USA; nkoziol@unl.edu; 10 Division of Biokinesiology and Physical Therapy, University of Southern California, 1540 E Alcazar Street, CHP 155, Los Angeles, CA 90033, USA

**Keywords:** cerebral palsy, Botulinum toxin-A, phenol, physical therapy, early intervention

## Abstract

Therapies for children with cerebral palsy (CP) often fail to address essential components of early rehabilitation: intensity, child initiation, and an embodied approach. Sitting Together And Reaching To Play (START-Play) addresses these issues while incorporating intensive family involvement to maximize therapeutic dosage. While START-Play was developed and tested on children aged 7–16 months with motor delays, the theoretical construct can be applied to intervention in children of broader ages and skills levels. This study quantifies the impact of a broader START-Play intervention combined with Botulinum toxin-A (BoNT-A) and phenol on the developmental trajectory of a 24 month-old child with bilateral spastic CP. In this AB +1 study, A consisted of multiple baseline assessments with the Gross Motor Function Measure-66 and the Assessment of Problem Solving in Play. The research participant demonstrated a stable baseline during A and changes in response to the combination of BoNT-A/phenol and 12 START-Play sessions during B, surpassing the minimal clinically important difference on the Gross Motor Function Measure-66. The follow-up data point (+1) was completed after a second round of BoNT-A/phenol injections. While the findings suggest the participant improved his gross motor skills with BoNT-A/phenol and START-Play, further research is needed to generalize these findings.

## 1. Introduction

Therapeutic interventions for young children with or at risk of developing Cerebral Palsy (CP) vary across early intervention service models [1] as limited evidence exists to guide practice [2]. In particular, service models in early CP rehabilitation often lack theoretical grounding [3] and concrete definitions of the essential components of early intervention [1]. Examples of limited definitions include the lack of information on the timing and dose of intervention; the role of the parent, therapist, and child in the intervention; and the primary focus of the intervention [1,3,4,5,6,7]. A recent systematic review of dosage of early intervention therapies reported that 119 hours over 3–10 weeks (11.9–40 h/week) was found to elicit improvements in motor outcomes in infants with CP [6]. However, early intervention therapies (provided under the Individuals with Disabilities Education Improvement Act [8]) average 2–3 h per month [5]. The under dosing of direct therapy services is justified by family engagement to provide adequate interventions outside of therapies within the context of family everyday routines [6,9,10,11]. Parents are thus expected to provide 9–38 h per week of activities to meet the dose guideline. In addition to the challenges of meeting adequate dosing, intervention may not promote movement driven by the infant’s cognitive curiosity, thus failing to be grounded in action-perception and embodied cognition theories [3,4]. Rather, many rehabilitation interventions focus simply on body structure and functions without adequately considering the multi-domain developmental changes necessary for integrating therapeutic procedures into a child and family’s daily activities [12,13]. 

A defining characteristic of spastic CP is increased velocity dependent stiffness or spasticity in select muscles, which limits movement variability and independent environmental exploration [14]. Medical interventions to reduce spasticity, such as Botulinum toxin-A (BoNT-A) [15,16], or BoNT-A and phenol [17,18], may temporarily reduce spasticity, increasing a child’s joint mobility and potential to explore their environment with greater movement variability [15,16,17]. Phenol nerve blocks administered to the tibial and obturator nerves of children with spasticity (ages 2 to 8+ years) have been shown to last 3–6 months, to improve creeping and standing patterns [19], and take effect almost immediately [20]. However, administration of phenol is complex and generally not well tolerated by children with CP in that anesthesia is often required for administration [18,19]. Numerous studies, with outcome measures performed around the peak effect time of BoNT-A (4-8 weeks after injections [21,22], lasting 3–6 months [23]), suggest that BoNT-A effectively reduces spasticity and improves passive and active range of motion in the muscles targeted by injection [24,25,26,27]. During peak effect, BoNT-A therapy also resulted in increased mobility as measured by the Gross Motor Function Measure and gait evaluations [25,27,28]. However, identifying key principles of the intervention needed to maximize the benefit of combined rehabilitation and BoNT-A/phenol is unclear, particularly in children under 2 years of age [15,16,29]. BoNT-A treatment in children under the age of 2 years remains controversial primarily due to a dearth of high-quality research [15,29]. This single subject research design (SSRD) was utilized to add to the literature examining the efficacy of combing BoNT-A and phenol with a targeted physical therapy intervention at a higher than typical dose. A novel physical therapy intervention, Sitting Together And Reaching To Play (START-Play) [30] was designed to facilitate increased variability in environmental exploration by motivating young children to independently engage in motor-based problem solving [30], making START-Play a suited intervention to compliment spasticity management with BoNT-A and phenol in young children with CP.

START-Play [30] is an evidence-based intervention which has well defined key intervention components and includes the integration of dynamic systems [31,32], perception action [33,34], and embodied cognition [35,36] theories to support child directed, motor based problem-solving [30]. This model was originally developed with efficacy evaluated when applied to the specific skills of sitting and reaching [30]. In a multi-site clinical trial [30], START-Play was found to be most effective for children with severe motor impairments, defined as >2.5 standard deviations below the mean on the motor composite scale of the Bayley Scales of Infant Development, 3rd Edition (BSID-III) [30,37]. START-Play utilizes four key cognitive concepts which support early motor-based problem solving: object permanence, means end, joint attention, and object and body affordances [38,39]. Learning these four concepts relies heavily upon foundational motor abilities (head control, reaching and grasping, sitting, and mobility) targeted by the START-Play intervention [30,39]. For example, objects for exploration are playfully hidden by the START-Play physical therapist to entice the child’s contingent reach and weight shift while reinforcing the child’s understanding of object permanence. Starting with a toy partially hidden and working toward a fully covered toy teaches object permanence and allows the primary caregiver, child, and therapist to attend jointly to the found object. Motor and cognitive challenges are scaled to just beyond the child’s current abilities and are specifically tailored to encourage the child to self-select the motor-based problem solving needed for individually salient play, mobility, and social engagement [30,39].

The START-Play intervention therapist’s focus on tailoring tasks escalating in difficulty and individualizing the environment to encourage self-generated and variable motor-based problem solving is essential for children with spasticity related to CP [1,3,4,6,40]. Incorporating START-Play during the time of maximum efficacy of BoNT-A/phenol has the possibility of increasing the use of newly available motor patterns for the child to explore, thus broadening the child’s experience with movement-based problem solving. However, the combination of these interventions has not been studied.

The SSRD presented is unique in that it combines a medically indicated spasticity management plan with a personalized therapeutic intervention based on the START-Play model. The SSRD was initially conceptualized with a multiple baseline design ideally suited for the temporary effects of spasticity reduction of BoNT-A [23,41] and phenol [17,18]. Additionally, using a SSRD allowed for accurate reporting regarding the effects of individualized treatments for children with characteristically heterogenic conditions such as CP [42,43]. The purpose of this SSRD was to explore possible changes in the target variables of motor and problem-solving outcomes associated with an individualized intervention plan including START-Play physical therapy intervention and BoNT-A/phenol administration in a 2-year-old male with bilateral spastic CP. We hypothesized that this individualized intervention would allow this child with bilateral spastic CP to overcome a plateau in motor skill development and improve his participation in his home and future school environments, even after the effects of BoTN-A and phenol dissipated.

## 2. Methods

### 2.1. Participant Description

The male participant, “S”, was born preterm at 32 weeks of gestation with a birth weight of 1300 g. His Neonatal Intensive Care Unit (NICU) stay lasted until 28 days after birth. Cranial ultrasound detected a grade 3 intraventricular hemorrhage, and magnetic resonance imaging confirmed chronic periventricular leukomalacia and bilateral germinal matrix hemorrhages reflecting a prenatal ischemic injury. S was diagnosed with bilateral spastic CP using the international guidelines for early detection of CP [44] at chronological age 13 months. At chronological age 24 months, S’s motor skill level was classified as consistent with Gross Motor Function Classification System (GMFCS) Level III by his physical therapist [45], indicating S sat independently, crept on his stomach or hands and knees via “bunny hopping” (lacking lower extremity dissociation), pulled to standing and initiated cruising at furniture, and walked with hand-held assistance or the use of an assistive device (gait trainer).

S received occupational therapy from his local early intervention program twice a month in his home. In addition, he received weekly outpatient physical therapy. Per his mother, therapy sessions focused on relaxation of spastic muscles, stretching activities, balance challenges, and the use of assistive devices, primarily flexible supra-malleolar orthotics, and a gait trainer. He had been prescribed compression garments for his lower extremities, trunk, and upper extremities by a physician. Family long-term therapy goals included S achieving independence with activities of daily living and mobility to enable full participation in his local school environment. After his 2-year-old visit with the NICU follow up program and spasticity clinic, BoNT-A injections with subsequent intensive physical therapy were recommended. The family’s goal for study participation was for S to transition from creeping on his hands and knees via a “bunny hop” to explore his home and community via walking. 

**Figure 1:**
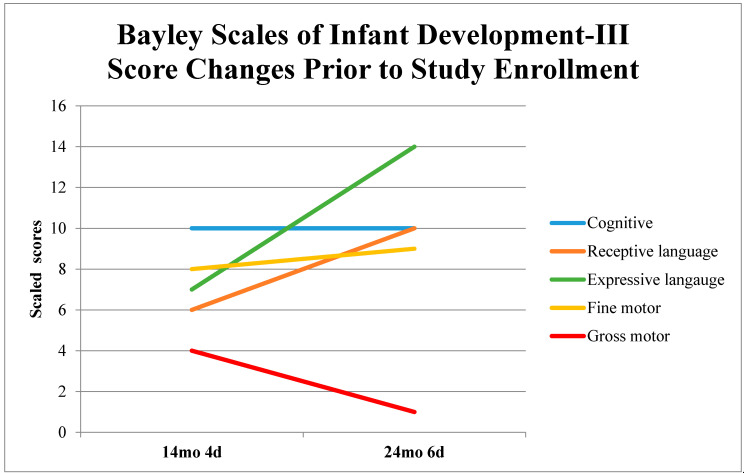
Bayley Scales of Infant Development-Third Edition Changes from 14 to 24 months Chronological Age. Changes in Bayley Scales of Infant Development, 3rd Edition, scores over 10 months as assessed at 14 and 24 months chronological age in NICU follow up clinic prior to study enrollment. mo = months, d = days. The scaled score population mean is 10 with a standard deviation equal to +/−3 [38].

S was determined to be a candidate for START-Play combined with BoNT-A/phenol treatment for a number of reasons. S’s development was monitored using the BSID-III [37] at the NICU follow-up clinic prior to study enrollment. The BSID-III is a widely used, norm-referenced test to assess the developmental functioning of children ages 1–42 months [37]. S’s medical records revealed a lack of progress in the gross motor domain, with a scaled score more than 2.5 SD below the mean (Figure 1). Children >2.5 standard deviations below the mean have been shown to optimally benefit from the START-Play intervention in a recent clinical trial [30]. S was also in the target age range the START-Play developers considered ideal for broadening of the START-Play intervention to cover development of more than just sitting and reaching. S had excellent fine motor and cognitive skills that were age appropriate (Scaled scores of 8 and 9 for fine motor and 10 and 10 for cognitive at 14 and 24 months of chronological age). S’s preference was to sit with his legs arranged in a “W”. In this “W”-sitting position, S would happily sit and play, challenging his cognitive and fine motor development, yet minimizing his need to utilize gross motor skills to explore. S’s expressive language increased from a scaled score of 7 at 14 months to 14 at 24 months of chronological age (raw score from 12 to 35, respectively). His receptive language scaled score increased from 6 at age 14 months to 10 at age 24 months of chronological age (raw scores from 11 to 24, respectively).

S’s family gave their informed consent for inclusion before they participated in the study. The study was conducted in accordance with the Declaration of Helsinki, and the protocol was reviewed and approved by the associated Institutional Review Board.

### 2.2. Study Design and Measures

An AB +1 SSRD [46] was utilized to evaluate the efficacy of a combined physical therapy (START-Play) and BoNT-A/phenol intervention. Primary outcome measures included the Assessment of Problem Solving in Play (APSP) [47] and the Gross Motor Function Measure-66 (GMFM-66) [48]. The APSP is a tool designed to sensitively quantify changes in problem solving over time for young children with and without motor impairments [47]. The APSP is a reliable test with interrater percent agreements of 83–100% and with high concurrent validity shown with the BSID-III in assessing motor-based problem solving of infants and young children with motor delays [47]. The GMFM-66 is an assessment tool designed to measure changes in gross motor development in children with CP over time or in response to intervention [48]. Strong psychometric properties have been demonstrated with the GMFM-66, such as a test–retest reliability with an intra-class correlation coefficient (ICC) of 0.9932 and strong construct validity between age and GMFCS levels [49]. Within the AB +1 design, A was a non-intervention phase, utilized for gathering data from multiple baseline measures. While not part of the official study, S’s NICU follow up clinic administered the BSID-III [37] (See Figure 1) and GMFM-66 [48] assessments which were completed as part of clinical care prior to his enrollment in this study (Table 1, columns 1 and 2). The initial GMFM-66 score noted in A was taken from the NICU follow-up clinic at age 24 months chronological age. The interrater reliability of the investigator who administered the GMFM-66 at the NICU follow up clinic has been established in previous studies [50]. With the exception of the first GMFM-66 assessment, all of the remaining GMFM-66 assessments were scored by a blinded assessor in randomized order with the dates removed. The primary assessor of the GMFM-66 has good to excellent interrater reliability as established in a larger START-Play clinical trial with ICCs of 0.8–0.98 [30,51]. As the NICU follow-up clinic does not routinely administer the APSP, one fewer APSP result was attained during A. The BSID-III assessments from the NICU follow-up clinic have been included as data gathered prior to A for reference of S’s rate of change for 10 months prior to the start of the study (See Figure 1). Based on the results of the BSID-III indicating a plateau in gross motor skill development and the desire for improvements in motor-based problem solving relating to the key ingredients of START-Play, target variables for this SSRD include gross motor skill level as measured by the GMFM-66 [48] and motor-based problem solving as measured by the APSP [47].

This combined BSID-III, GMFM-66, and APSP data were utilized to individualize the START-Play intervention in B. B was the intervention period which started with the administration of BoNT-A/phenol. To allow adequate time for the BoNT-A/phenol to decrease S’s spasticity [21,22,23], the START-Play intervention was initiated 2 weeks after the injections. During B, START-Play was provided 2 times per week with each session lasting approximately 60 min. The planned 12 weeks of START-Play was ended after 6 weeks due to Covid-19. BoNT-A/phenol injections without START-Play were administered approximately 4.5 months after the initial injections. A telemedicine assessment was completed 12 weeks after the second round of injections, known as the “+1” data point, providing some information on the change with BoNT-A/phenol alone and allowing for an AB +1 study model. 

### 2.3. Interventions 

#### 2.3.1. Botulinum Toxin-A and Phenol

Prior to the administration of BoNT-A and phenol at the age of 26 months, S presented at the age of 25 months with a Modified Ashworth Scale (MAS) [52] score of 3, indicating a considerable increase in muscle tone rendering passive range of motion difficult into bilateral hip abduction, knee flexion, knee extension, and ankle dorsiflexion. Oral Baclofen, 2.5 milligrams, two times per day was initiated and continued throughout the SSRD time frame. Prior to BoNT-A and phenol administration at age 26 months and with oral Baclofen, MAS scores had only decreased to 2–3 in the tracked muscle groups. Due to the patient’s presentation of a crouched standing position with weight bearing primarily on the toes and relatively stable MAS scores, individualized BoNT-A/phenol injections were administered while sedated with propofol to target key muscle groups. The anterior branch of the right obturator nerve was localized under ultrasound, lying deep to the adductor longus on the surface of the adductor brevis. A 1.5-inch, 26-guage needle was advanced under ultrasound guidance into the proximity of the targeted right anterior obturator nerve. Electrical stimulation was delivered through the needle, prompting an adductor muscle twitch, which confirmed accurate placement. 0.5–1.0 cubic centimeters (cc) of phenol was injected into 4 sites along the right anterior obturator nerve. The same procedure was repeated on the left side. A total of 3cc of phenol was injected on each side. Using ultrasound guidance with visual confirmation of needle placement, S was injected with a total of 120 units (10 units/kilogram) of BoNT-A into the following muscles: right and left semimembranosus (25 units each); right and left biceps femoris long head (15 units each); right and left medial gastrocnemius (10 units each); and right and left lateral gastrocnemius (10 units each). At age 27 months, MAS scores had changed to 1–2 with movement into bilateral hip abduction, knee flexion, and knee extension, and 2 into ankle dorsiflexion (indicating a decrease in spasticity to slight to marked increase in muscle tone throughout most of the range of motion) [52]. A second round with identical dosages and injection techniques of BoNT-A and phenol was initiated after S’s outpatient physical therapist notified the physician of a significant increase in muscle tone at age 30.9 months with MAS scores returning to 3 throughout, 4.5 months after the initial injection [53,54]. 

#### 2.3.2. START-Play

**Figure 2:**
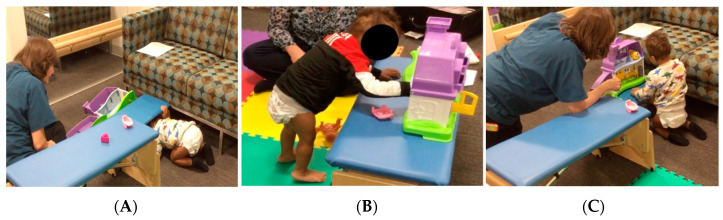
Sitting Together And Reaching To Play (START-Play) Intervention. When S played with a toy house, he needed to problem solve the best solution for play with the smaller toy house pieces. He could decide if (**A**) he wanted to transition from an upright posture to a lower one to look under the bench for hand-held toys, (**B**) he wanted to cruise around the bench to access a different affordance of the toy, or (**C**) he wanted to make a postural adjustment, such as transitioning from heel sitting to tall kneeling, to better visually engage with the toy house to manipulate and role play a story with the smaller toy pieces (affordance). S chose the toy house to play with which provided a variety of combined motor and problem-solving opportunities. He self-selected the best motor solution for the play task with his parent or therapist simultaneously supporting the “just right” level of challenge via environment and task modifications.

The START-Play intervention was individualized for S specifically targeting transitions between lower postures (sitting and prone lying) to more upright postures (standing, kneeling, walking, and climbing) for this child who was cognitively and socially engaging and explored his immediate environment primarily through “bunny hopping”. During transitional movements, S was provided with play activities designed to challenge spatial memory while also exploring object affordances and means ends tasks. Support for both cognitive and motor skills was provided to deliver the “just right” challenge level of the activity allowing S to advance to higher developmental levels (Figure 2).

For each skill the child practiced during the START-Play sessions, the physical therapist tailored escalating challenges in both motor and cognitive areas of development. The increments of change were small enough to keep S engaged and motivated, while expanding his movement and problem-solving repertoire. Due to the distance between the participant’s home and the study site, the START-Play intervention was delivered in a clinic environment located within the research laboratory. 

### 2.4. Statistical Analysis

Data were analyzed using the two-standard deviation band (2SDB) method [46]. This method was selected due to sensitivity to detect the anticipated small magnitude treatment change over the short period of time [46]. Consistent with the 2SDB method, the mean and standard deviation of the A outcome measure scores (GMFM-66 and APSP) were calculated. The score at each timepoint in phase B (treatment phase) was compared with the mean and the 2SDB of A. Gottman and Leiblum [55] state that if two or more consecutive data points fall outside of the 2SDB range, a significant change in skill development has occurred. The data were analyzed to determine the direction of the change (above or below the 2SDB range) and the number of points outside of the 2SDB range. 

## 3. Results

**Figure 3:**
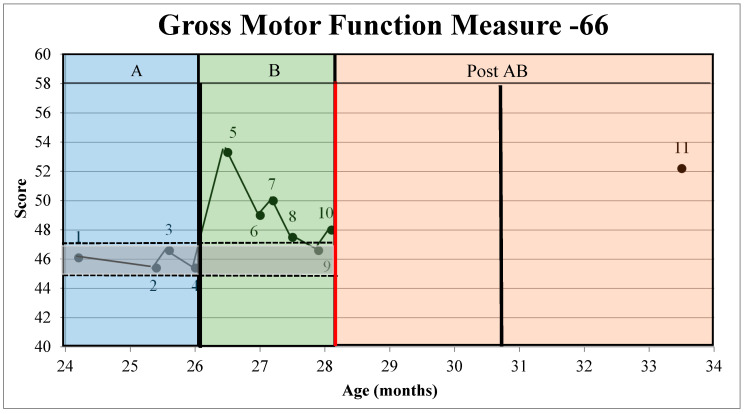
Gross Motor Function Measure Across the AB +1 Phases. Scores of the Gross Motor Function Measure (GMFM-66) across the AB +1 phases as plotted in reference to the +/− 2 standard deviation band (shaded in grey). A = baseline data collection with no intervention, B = Botulinum toxin A/phenol and START-Play physical therapy intervention provided for 12 sessions, Post AB = one data point collected via telemedicine (+1) due to Covid-19 restrictions. Points 1–11 denote GMFM-66 testing points. Bolded, black, vertical lines denote Botulinum toxin-A/phenol injections. The bolded, red vertical line denotes treatment ending due to the Covid-19 pandemic.

**Figure 4:**
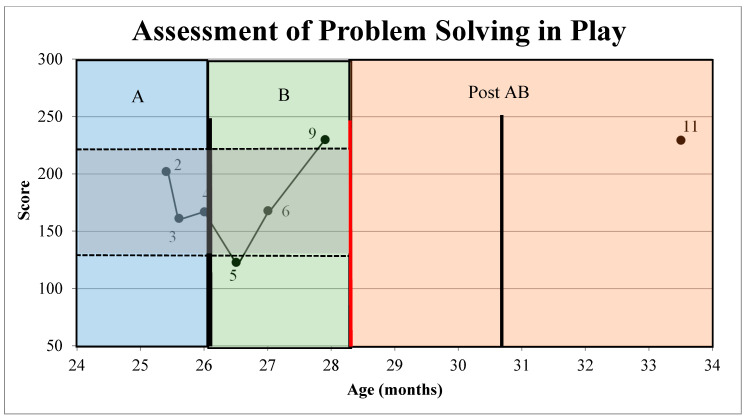
Assessment of Problem Solving in Play Across the AB +1 Phases. Scores of Assessment of Problem Solving in Play (APSP) (reported in frequency APSP/minute) across the AB +1 phases as plotted in reference to the grey shaded +/− 2 standard deviation band. A = baseline data collection with no intervention, B = Botulinum toxin A/phenol and START-Play physical therapy intervention provided during data collection, Post AB = data point 11 collected via telemedicine (+1). Points 2–9 and point 11 represent administered APSP data points. Points 1, 7, 8, and 10 were omitted from the figure as these data collection days did not include APSP testing due to testing site or child-related limitations. Bolded, black, vertical lines denote Botulinum toxin-A/phenol injections. The bolded, red vertical line denotes treatment ending due to the Covid-19 pandemic.

On both the GMFM-66 [48] and APSP [47], relatively stable baselines were documented (Figure 3 and Figure 4) in A, providing an appropriate 2SDB to measure change in phase B of the AB +1 design. The A averages of the 4 GMFM-66 [48] scores were 45.88 (standard deviation or SD = 0.59). Thus, any GMFM-66 [48] score over 47.02 was above the 2SDB (See Figure 3). Likewise, the mean of the 3 APSP [47] scores was 177 (SD = 22.11), making any score over 221 above the 2 SDB, representing a measurable change (See Figure 4). 

The 5 GMFM-66 [48] timepoints in B averaged to 48.22 (SD 1.33), which is greater than the upper limit of the 2SDB (47.1, as established in A) and is sufficient to indicate a significant change in S’s gross motor outcome measure [55]. Five out of the six B points (See Figure 3) on the GMFM-66 were above the 2SDB, thus, indicating a significant change has occurred with the combined intervention of BoNT-A/phenol and START-Play as measured by the GMFM-66 [48]. Additionally, the minimal clinically important difference (MCID) for a child at GMFCS Level III [45] is indicated by a change of GMFM-66 scores of 1.2 with a large (0.8) effect size [56]. The difference between the mean GMFM-66 score of A (45.88 mean score) and B (48.22 mean score) was 2.34, indicating a clinically significant change in gross motor function when comparing the means of A and B. 

The average B APSP [47] score was 174 (SD = 26.8), which is not greater than the upper limit of the 2SDB band of 221 (Figure 4). Additionally, there were not 2 consecutive data points with scores over the 2SDB. Both of these analyses suggest no change in APSP [55]. Data on the MCID of the APSP are not yet available. 

The scores for the +1 visit were 52.2 for the GMFM-66 [48] and 229.5 for the APSP [47]. No comparison between A, B, and the +1 telemedicine visit can be made as a new baseline was never established by performing repeated assessments to determine a new mean and 2SDB band. 

## 4. Discussion

The combination of BoNT-A/phenol and targeted physical therapy using the START-Play approach appears to have helped S overcome the plateau in his gross motor skills while continuing to support his problem solving. In the transition between A and B on the GMFM-66 (See Figure 3), there is a substantial jump in GMFM-66 scores of 45.4 to 53.3. This score difference is most likely explained by the decrease in spasticity combined with testing variability in this 2-year-old child. While one data point (Point 9) (See Figure 3) dipped below the 2 SDB, it is not clear if S’s skills were regressing toward the baseline average established in A. The decrease in GMFM-66 scores at Point 9 may have been related to the waning effects of BoNT-A/phenol, as expected, with a 4–8 week peak in effectiveness following BoNT-A injections [21,22]. However, spasticity management related to the effectiveness of phenol nerve blocks has been reported to last for 3–6 months [19]. After BoNT-A/phenol, S was able to explore his environment with greater ease as represented by the GMFM-66 scores, likely as a result of decreased spasticity and improved range of motion [16]. The BoNT-A/phenol thus may have served as a primer to maximize the benefits as S entered the START-Play segment of treatment. Taking advantage of the potential priming, S entered a stage of intense, self-generated, and variable motor exploration facilitated by an intervention designed to enhance his self-directed mobility and problem-solving [1,57].

This SSRD demonstrated an individualized intervention including medical intervention and physical therapy based on current developmental theory with well-defined intervention principles [3,30]. Providing S with the “just right” cognitive and motor tasks allowed him the ability to explore the environment, seek his own solutions to motor-based problem solving, and decipher new motor patterns. Applying individualized interventions based on perception-action theory requires physical therapists to seek advanced training to successfully employ multi-domain interventions [3,30,58]. Previous research denotes the efficacy of similar perception-action based motor interventions [3,34,57] such as Goals-Activity-Motor Enrichment (GAME) [59] and Supporting Play Exploration and Early Development Intervention (SPEEDI) [60] as interventions appropriate for a physical therapist to facilitate motor skill development in children with or at high risk of developing CP. The broadened version of the START-Play intervention combined with BoNT-A/phenol provided a multi-domain, individualized, evidence-based intervention for S to establish a significant change in his gross motor outcome at a young age, taking advantage of early neural plasticity [15,16,26,61].

The START-Play intervention additionally includes a focus on timely responsiveness from parents which increases the quantity and quality of “serve and return” [7] in parent–infant relationships. We believe START-Play’s focus on quality parent–child interaction allows the parent to develop the knowledge and sensitivity to provide the “just right” challenge during parent-guided home interventions, both between START-Play therapy sessions and upon completion of the START-Play intervention. The focus on parent engagement directly carries over to a wide variety of unique home environments. Enhancing both the physical [6] and social-emotional environment [7,62] to promote motor-based problem solving is noted as a contributing component of successful early intervention for children with CP [4].

Despite the success of BoNT-A/phenol combined with START-Play in demonstrating positive changes in developmental indicators, this SSRD has limitations. Due to the distance between the participant’s home and the study center, it was not feasible for the START-Play intervention to be delivered in the home environment. While START-Play can be delivered in a clinic or daycare setting, the home environment is the optimal setting [30]. Future research using START-Play should ideally be performed in the home environment. During the administration of the APSP [47], the study participant’s compliance with sitting and manipulating toys was challenged as attested by the number of incomplete APSP assessments (See Table 1 and Figure 4). While administering the APSP, it is possible that participation would have been improved if the APSP allowed for more engaging toys based on the participant’s interest, such as a toy house instead of the standardized nesting cups [47]. Additionally, the APSP generally was administered after the GMFM-66. Reversing the order of the tests may have improved participation. We were not able to isolate the effects of BoNT-A/phenol or START-Play or repeat the planned second cycle of baseline and intervention assessment/treatment. These factors severely limit the ability to interpret this study from a causality perspective and limit our ability to fully interpret our hypotheses [43,63]. Ultimately, our study design was changed from ABAB to AB +1. In addition, the B cycle was shorter than we generally recommend for a burst of intervention. Previous researchers provided the START-Play intervention for 12 weeks with 2 sessions per week [30]. Thus, only 50% of the typical START-Play intervention was provided to this child. It is unclear at this time if increased intervention would have significantly impacted the resulting number of timepoints above the 2SDB on either the APSP or GMFM-66. Future research is needed with more rigorous research designs. To discern causality from a SSRD, a minimum of 3 return-to-baseline cycles are recommended [43,63]. With 3 or more baseline data collections, we would expect to see a successive elevation in baselines, reflecting the ongoing impact of primary caregiver training and the embodied motivation of the participant to engage in motor-based problem solving.

## 5. Conclusions

This study adds to the literature regarding potentially effective, evidence-based, individualized interventions for children with CP provided before 3 years of age. Exploring BoNT-A/phenol combined with START-Play attempts to satisfy the need for further studies that explore the use of BoNT-A/phenol in young children and the need to facilitate exploration of interventions in combination with BoNT-A/phenol for young children with CP [15,29]. Of great importance for S and his family, the combination of BoNT-A/phenol and START-Play intervention appears to have improved upon motor-based problem solving and likely assisted in overcoming a plateau in gross motor skill development seen between 12 and 24 months of age in this child.

## Figures and Tables

**Table 1:** Study Phases with Chronological Age and Data Collected.

Phase	Datapoint	Chronological Age (mo)	GMFM-66	APSP
A	1	24.2	46.1	X
	2	25.4	45.4	202
	3	25.6	46.6	161
	4	26	45.4	167
B	5	26.5	53.3	123
	6	27	49	168
	7	27.2	50	X
	8	27.5	47.5	X
	9	27.9	46.6	230
	10	28.1	48	X
**COVID-19 paused visits**				
Post AB	11	33.5	52.2	229.5

Phase description of the AB +1 single subject research design. A= baseline data collection with no intervention, B = Botulinum toxin A/phenol and Sitting Together And Reaching To Play (START-Play) physical therapy intervention provided during data collection, Post AB = Botulinum Toxin A/phenol intervention only with one data point collected via telemedicine (+1). GMFM-66 = Gross Motor Function Measure-66 summary scores, APSP = Assessment of Problem Solving in Play results in frequency APSP/minute. X = no data collected.
